# 2-Hydroxymethyl-1-methyl-5-nitroimidazole, one siderophore inhibitor, occludes quorum sensing in *Pseudomonas aeruginosa*

**DOI:** 10.3389/fcimb.2022.955952

**Published:** 2022-09-08

**Authors:** Lujun Yin, Wang Shen, Jun-Sheng Liu, Ai-Qun Jia

**Affiliations:** ^1^ School of Pharmaceutical Sciences, Key Laboratory of Tropical Biological Resources of Ministry of Education, Hainan University, Haikou, China; ^2^ One Health Institute, Hainan University, Haikou, China

**Keywords:** 2-hydroxymethyl-1-methyl-5-nitroimidazole, siderophore inhibitor, quorum sensing, virulence factor, biofilm, *Pseudomonas aeruginosa*

## Abstract

Siderophore is necessary for the survival of microorganisms and is interregulated with quorum sensing (QS) systems. It is related to growth, proliferation, virulence, and other bacterial social activities as a virulence factor. Thus, we speculated that the QS system could be occluded by inhibiting siderophore production. 2-Hydroxymethyl-1-methyl-5-nitroimidazole (HMMN), one siderophore inhibitor of *Pseudomonas aeruginosa* PAO1 (*P. aeruginosa* PAO1), was obtained by using the Chromeazurol S (CAS) method. We found that HMMN inhibited siderophore production and influenced the biological effects of QS regulation, including biofilm formation and pyocyanin production. HMMN (150 μg/ml) inhibited the siderophore production of *P. aeruginosa* PAO1 by 69.37%. In addition, HMMN could inhibit pyocyanin production and biofilm formation and erase the formed biofilm of *P. aeruginosa* PAO1. HMMN (150 μg/ml) inhibited the biofilm formation of *P. aeruginosa* PAO1 by 28.24%. The erasure rate of the formed biofilm reached 17.03%. Furthermore, HMMN (150 μg/ml) inhibited *P. aeruginosa* PAO1 pyocyanin production by 36.06%. Meanwhile, positive-control hordenine (500.0 μg/ml) reduced the biofilm formation and pyocyanin production of *P. aeruginosa* PAO1 by 14.42% and 34.35%, respectively. The erasure rate of hordenine to the formed biofilm is 11.05% at 500 μg/ml. Quantitative real-time polymerase chain reaction (qRT-PCR) showed that HMMN downregulates not only siderophore-related genes but also QS-related genes, as well as hordenine. These results suggest that a siderophore inhibitor could be used as a QS inhibitor to occlude the QS system and reduce virulence.

## Introduction

Iron is one crucial nutrient factor for the survival of most organisms on earth. However, most of the iron in the environment is in the form of compounds with low free iron concentrations due to the presence of oxygen, and very low amounts of iron are used for microbial growth requirements (Haque et al., 2018). Pathogens face a severe iron uptake problem in their hosts. They have evolved into a series of systems that could chelate and uptake iron (siderophore) to maintain their growth and perform various physiological functions (Lu et al., 2021).

3,4-(Di)hydroxy-heptyl-2-quinolone (PQS) acted as one quorum sensing (QS) signal molecule to control the siderophores’ (pyochelin and pyoverdine) synthesis gene expression in *Pseudomonas aeruginosa* (*P. aeruginosa*) (McLean et al., 2004). It indicates that the QS system has a wide range of regulatory effects on siderophore-related genes. It was found that there is an extensive connection between siderophores and the QS system, and they jointly regulate the biological effects of microorganisms, including virulence factors, biofilm formation, and other physiological processes of bacterial infection (Zhou et al., 2013). Abbas et al. found that *ton*B and *tol*QRA genes played a significant role in iron transport and were both associated with QS (Wang et al., 2011). Alain Stintzi et al. found that the *lasR* mutant reduced the siderophore production of *P. aeruginosa* (Stintzi et al., 1998). Fumiko Taguchi et al. reported that *pvdJ* and *pvdL* mutants, siderophore biosynthesis defects in *P. syringae*, showed lower abilities to produce tabtoxin, acyl-homoserine lactones (AHLs), and extracellular polysaccharide (EPS) and reduced virulence on host tobacco plants (Taguchi et al., 2010). In addition, some QS signals can chelate iron to maintain growth. The QS signal PQS has an Fe^3+^ chelating function (Xu et al., 2017). Two siderophores, pyochelin and pyoverdine, also play the QS signal molecule roles in *P. aeruginosa* (Gu et al., 2020).

The drug resistance of pathogenic bacteria, which is a serious problem, has received widespread attention. The drug-resistant strains are gradually increasing in number, and the tolerance range is gradually expanding (Woodford et al., 1997). In recent years, the “superbugs” of resistance to various commercial antibiotics have appeared worldwide (Andersson and Hughes, 2010; Olaru et al., 2021). Notably, accumulating evidence has demonstrated that some microbial resistance mechanisms are regulated by QS systems (Zhao et al., 2020). Thus, the blocking of the QS system could be used as a target to disrupt the communication system within a nuisance bacterial colony. QS inhibitors (QSIs) are substances that interfere with the QS systems. QSIs can interfere with biofilm formation, erase the formed biofilm, and inhibit virulence factors (Liu et al., 2017). QSIs can also be used in combination with traditional antibiotics to recover the sensitivity of traditional antibiotics against drug-resistant bacteria (Tabraiz et al., 2021). The discovery of the QSIs provides a robust method to reduce the high virulence and resistance of bacteria. Based on the relationship of the mutual regulation between siderophores and QS systems, the current study found that one siderophore inhibitor of *P. aeruginosa* could be used as a QSI to inhibit biofilm formation and pyocyanin production and erase the formed biofilm regulated by pathogenic bacteria QS. This work provides a strategy in which the siderophore inhibitor was used to prevent and treat drug-resistant chronic infections induced by QS.

## Materials and methods

### Bacteria strain, culture medium, and chemicals

Chromeazurol S (CAS), hexadecyl trimethyl ammonium bromide (CTAB), and resveratrol were purchased from Shanghai Macklin Biochemical Technology Co., Ltd. (Shanghai, China). Glucose, peptone, NaH_2_PO_4_, KH_2_PO_4_, and FeCl_3_ were purchased from Tianjin Damao Chemical Reagent Factory (Tianjin, China). MgSO_4_·7H_2_O, CaCl_2_, Na_2_HPO_4_·12H_2_O, NaCl, NH_4_Cl, MeOH, and 95% EtOH were obtained from Xilong Science Co., Ltd. (Guangzhou, China). The phosphate buffer solution (PBS) and dimethyl sulfoxide (DMSO) were acquired from Biosharp (Anhui, China). Agar powder was bought from Guangdong Huankai Microbial Technology Co., Ltd. (Guangdong, China), and N-methyl-3-phenyl-2-propen-1-amine was bought from Sigma (MO, USA). (5Z)-4-bromo-5-(bromomethylene)-2(5H)-furanone was kindly presented by Prof. W.M. Chen (Jinan University, China). Hordenine, 2-hydroxymethyl-1-methyl-5-nitroimidazole (HMMN), and crystal violet were obtained from Aladdin Biochemical Technology Co., Ltd. (Shanghai, China). Furthermore, *P. aeruginosa* PAO1 was obtained from Prof. Q.H. Gong (Ocean University of China, China).

### The preparation of Chromeazurol S medium and reagent

The CAS method was used to quantitatively analyze the siderophore content with slight modifications (Bethke et al., 2016). Firstly, glucose (100.0 g), peptone (20.0 g), MgSO_4_·7H_2_O (0.5 g), CaCl_2_ (0.5 g), and agar powder (20.0 g) were accurately weighed and then dissolved in 1 L of ultrapure water to obtain a solid medium. Subsequently, Na_2_HPO_4_·12H_2_O (12.1 g), NaH_2_PO_4_ (3.0 g), KH_2_PO_4_ (0.4 g), NH_4_Cl (1.3 g), and NaCl (0.6 g) were dissolved in ultrapure water (appropriate amount) and diluted to 500 ml to obtain a phosphate buffer (0.1 mol/L, pH = 6.8). In addition, the CAS solution was prepared by dissolving 0.7 g CAS in ultrapure water (500 ml). FeCl_3_ (0.27 g) and HCl (12 M, 0.83 ml) were selected to be dissolved in ultrapure water (appropriate amount), and then, the volume is ixed thoroughly and constant at 1 L. CTAB (1.5 g) was dissolved in ultrapure water (appropriate amount), and the volume is constant at 1 L. Finally, the CAS solution (500 ml) and FeCl_3_ solution (100 ml) were mixed and then slowly added to the CTAB solution (400 ml) and shaken to mix evenly to obtain a CAS reagent. Furthermore, the solid medium, phosphate buffer, and CAS reagent were sterilized at an autoclave of 121°C for 15 min. The preheated 60°C phosphate buffer (10 ml) and CAS reagent (10 ml) were added to the solid medium (80 ml, 60°C) and mixed evenly without generating bubbles and then poured into the plate.

### Siderophore quantitative analysis

The siderophore content of *P. aeruginosa* PAO1 was measured using a CAS reagent (Xu et al., 2021) with minor modifications. Firstly, the CAS reagent was prepared according to the above step. The siderophore content of *P. aeruginosa* PAO1 cultured for 0–72 h was determined. The cultured solutions (37°C, 180 *rpm*) of *P. aeruginosa* PAO1 were taken at an interval for 8 h in a sterile environment. Then, the cultured solutions were centrifuged at 10,000 rpm and 4°C for 5 min to gain the supernatant. The supernatant (1 ml) was mixed with the CAS reagent (1 ml) after throwing a 0.22-μm filter membrane and incubated for 1 h in the dark at room temperature. The absorbance of the reaction solution was tested at 680 nm by reading the microplates BioTek Instruments, Inc. (Vermont, USA) and recorded as *A_2_*. The culture medium without *P. aeruginosa* PAO1 was taken as the blank control and recorded as *A_1_*. The siderophore content (*SU*) was calculated by using the following formula:


$$SU\;\left(\%\right)=\;\frac{A_{1}-A_{2}}{A_{1}}\;X\;100\%$$

The effect of the siderophore content was determined for *P. aeruginosa* PAO1 treated with hordenine and HMMN. Ten microliters of different concentrations of hordenine (100, 80, 50, and 25 mg/ml) and HMMN (15, 10, and 5 mg/ml) were respectively taken and added into the bacterial suspension (1 ml, 10^4^–10^5^ colony forming units (CFU)/ml). The final concentrations of hordenine were 1,000, 800, 500, and 250 μg/ml, and the final concentrations of HMMN were 150, 100, and 50 μg/ml, respectively. The siderophore content of the prepared *P. aeruginosa* PAO1 bacterial suspension was measured according to the above method. The bacterial suspension without hordenine or HMMN treatment was taken as the blank control. The siderophore inhibition rate by hordenine or HMMN was calculated by using the following formula:


$$Y\;\left(\%\right)\;=\;\frac{SU_{1}-SU_{2}}{SU_{1}}$$

where *Y* (%) is the drug’s inhibition of siderophore production; *SU*_1_ is the amount of siderophores produced in the blank group; and *SU*_2_ is the siderophore generated after the drug treatment.

### Inhibition of biofilm formation

The inhibition of biofilm formation was measured according to the reported methods (Nazik et al., 2020) with minor modifications. First, the solution of *P. aeruginosa* PAO1 cultured for 16 h was diluted to a bacterial suspension (10^4^–10^5^ CFU/ml). In total, 10 μl of the HMMN solution of different concentrations (15, 13, 10, and 5 mg/ml) was added into 1 ml of the diluted bacterial suspension. Then, a mixed solution (200 μl) was added to a 96-well plate (Corning, New York, USA) and incubated at 37°C for 24 h, after which the overnight culture suspension was removed, washed three times with PBS, and oven-dried at 60°C. Subsequently, the biofilm was fixed for 15 min by using 200 μl of MeOH. After the methanol completely evaporated to dryness, 200 μl of crystal violet (0.05%) was added to each well to stain and was then removed after 15 min. The stained biofilm was washed three times with PBS and dried. Lastly, EtOH (95%; 200 μl) was used for decolorization and placed on a shaker at 180 rpm to decolorize for 15 min. The OD was performed by spectrophotometry at 570 nm.

### Removal of biofilms

The work solution (10^4–^10^5^ CFU/ml, 200 μl) was added to each well of the 96-well plate and incubated at 37°C for 24 h. The overnight culture was then removed, and the biofilm was washed three times with PBS. Subsequently, different concentrations of HMMN were added to fresh Luria–Bertani (LB) broth and mixed well. The mixed medium was taken (200 μl) and added to a 96-well plate with culture at 37°C for 24 h. The supernatant was removed after completing the culture. The quantification analysis of biofilms was performed according to the inhibition of the biofilm formation section. In addition, hordenine was used as a positive control.

### Determination of pyocyanin

The content of pyocyanin was measured by Pejcic et al.’s method (Abbas et al., 2007). The prepared drug solutions (HMMN, 150, 130, 100, and 50 μg/ml; hordenine, 1,000, 800, 500, and 250 μg/ml) were added to the *P. aeruginosa* PAO1 solution (10^4^–10^5^ CFU/ml), cultured at 37°C and 180 rpm for 16–24 h, and then centrifuged (4°C and 10,000 rpm, 5 min). A total of 5 ml of the supernatant was mixed with 3 ml of chloroform (Aladdin, Shanghai, China) and set aside for layering. Subsequently, the organic phase was collected and mixed with HCl (1 ml, 0.2 M) (Saiguo Biotechnology Co., Ltd., Guangzhou, China) and centrifuged at 4°C and 10,000 rpm for 5 min. In the end, 200 μl of the upper-layer solution was taken, and the OD_520_ was measured.

### Expression study of the isolation RNA by quantitative real-time PCR

The qRT-PCR was employed to evaluate the gene expression levels of QS- and siderophore-related genes. Total RNA was extracted using an RNA Extraction Kit (Sangon Biological Technology Co., Ltd., Shanghai, China) following the manufacturer’s instruction. Hordenine and HMMN were added to the bacterial solution (10^4^–10^5^ CFU/ml) and then incubated for 16–24 h at 37°C and 180 rpm. DMSO was selected as the negative control. The bacterial cell was collected through centrifuging (4°C and 12,000 *rpm*, 15 min) after cultivation. Then, 100 μl of lysozyme (400 μg/ml) was added and shaken to mix well, and 900 μl of Buffer Rlysis-B (RNA Extraction Kit) was immediately added for 3 min at room temperature. Subsequently, 200 μl of chloroform was added and mixed thoroughly and then centrifuged at 4°C and 12,000 rpm for 15 min to remove the supernatant. Anhydrous ethanol (1/3) was added to the supernatant and centrifuged at 12,000 rpm and 4°C for 5 min after leaving it for 3 min to obtain the precipitate. The precipitate was washed using 700 μl ethanol (75%), and the supernatant was discarded after centrifugation (4°C and 12,000 rpm for 3 min), which was repeated once. The centrifuge tubes were inverted at room temperature for 10 min to allow for complete ethanol evaporation. Then, DEPC-treated ddH_2_O (30–50 μl, RNA Extraction Kit) was added to dissolve the precipitate and stored at -70°C before use. A quantitative real-time polymerase chain reaction (qRT-PCR) was carried out with an ABI 7300 Plus real-time PCR system. The amplification was carried out in 20 μl of reaction volume containing 2 × T5 Fast qPCR Mix (SYBR Green I; 10 μl, Tsingke Biotechnology, Beijing, China), primers (0.8 μl of each), diluted cDNA (1 μl), and ddH_2_O (7.4 μl). The thermocycling conditions were as follows: incubation for 10 min at 95°C, followed by denaturation for 15 s at 95°C and annealing and extension at 60°C for 60 s. PCR amplification consisting of 45 cycles was conducted. All samples were run in triplicate. The sequences of the primers of target genes are listed in **Table S1**. In addition, 16S rRNA served as the internal control. The relative expression levels of target genes were quantified by the 2^-ΔΔCt^ method.

In addition, to further interpret the relationship between siderophore- and QS-related genes, heatmap analyses were performed using the online analysis platform Hiplot (https://hiplot.com.cn/basic/heatmap).

### Statistical analysis

Mean values ± standard deviation (± SD) were used to represent the data. Statistical analysis was carried out using Graphpad Prism 8 software (San Diego, CA, United States). A one-way ANOVA plus *post hoc* Tukey test was used to evaluate the statistical significance between groups. Differences were considered statistically significant at *p< 0.05, **p< 0.01, and ***p< 0.001.

## Results

### Effects of the reported quorum sensing inhibitors against *P. aeruginosa* PAO1 siderophore

**Figure 1:**
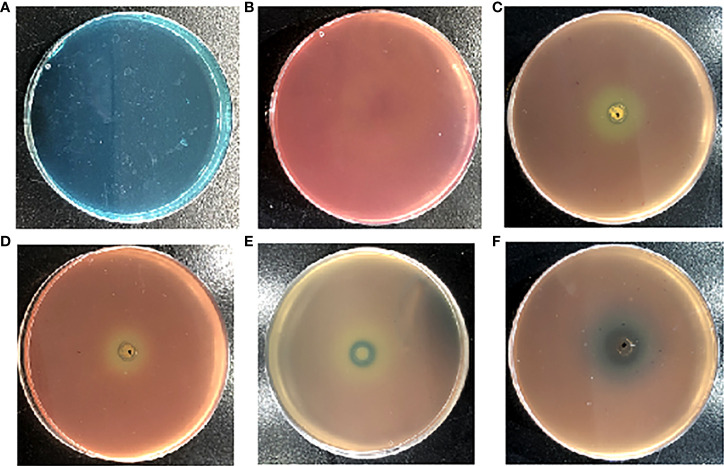
The effects of the different reported quorum sensing inhibitors (QSIs) on *Pseudomonas aeruginosa* PAO1 in Chromeazurol S (CAS) assays. The growth state of *P. aeruginosa* PAO1 in the CAS method **(A)**. The CAS method of pathogen-free growth **(B)**; hordenine **(C)**; resveratrol **(D)**; N-methyl-3-phenyl-2-propen-1-amine **(E)**; and (5Z)-4-bromo-5-(bromomethylene)-2(5H)-furanone **(F)**.

Siderophore inhibitors were obtained by using the CAS method. The principle is as follows. The original color of the CAS plate is blue green (**Figure 1A**). The bacteria containing siderophores can chelate the iron from the CAS plate with bacterial growth, leading to a plate color change from blue green to orange yellow (**Figure 1B**). Bacteria do not chelate iron from the plate when one compound inhibits siderophore production. The plate keeps the color blue green, while it cannot change to orange yellow.

In addition, the current study found that the reported *P. aeruginosa* PAO1 QSIs (hordenine, resveratrol, N-methyl-3-phenyl-2-propen-1-amine, and (5Z)-4-bromo-5-(bromomethylene)-2(5H)-furanone) can also inhibit siderophore production through the CAS method (**Figures 1C–F**). **Figure 1C** shows that hordenine, one reported QSI, can inhibit the siderophore production of *P. aeruginosa* PAO1. Another two QSIs (resveratrol and (5Z)-4-bromo-5-(bromomethylene)-2(5H)-furanone) can also inhibit the siderophore production of *P. aeruginosa* PAO1 (**Figures 1D, F**). Chen et al. demonstrated that (5Z)-4-bromo-5-(bromomethylene)-2(5H)-furanone can inhibit biofilm formation by blocking the QS system (Zhou et al., 2017). Ren et al. found that (5Z)-4-bromo-5-(bromomethylene)-2(5H)-furanone (QSI) can inhibit the siderophore biosynthesis of *P. aeruginosa* (Pejcic et al., 2020). The current study found that N-methyl-3-phenyl-2-propen-1-amine, a newly reported QSI against *P. aeruginosa* PAO1 in our group (Zhou et al., 2018), can also inhibit the siderophore production of *P. aeruginosa* PAO1 (**Figure 1E**).

### Screening of one siderophore inhibitor (2-hydroxymethyl-1-methyl-5-nitroimidazole) by Chromeazurol S method

**Figure 2:**
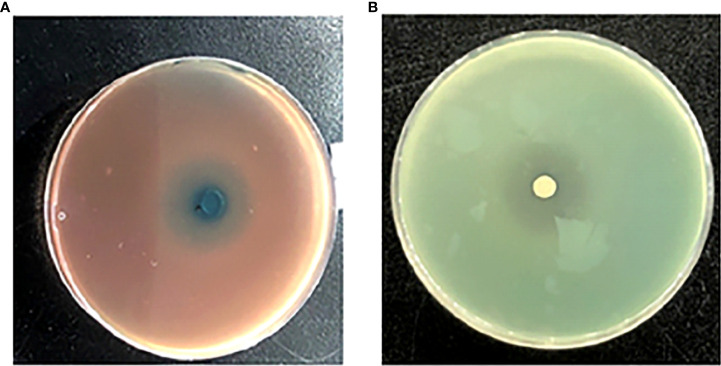
The influence of 2-hydroxymethyl-1-methyl-5-nitroimidazole (HMMN) on the growth of *P. aeruginosa* PAO1 in Luria–Bertani (LB) broth and the CAS assay. CAS assay **(A)** and LB broth **(B)**.

One siderophore inhibitor (HMMN) against *P. aeruginosa* PAO1 was obtained using the CAS method (**Figure 2A**). It showed that HMMN is surrounded by blue green in the CAS assay plate, which indicates that it has an inhibitory effect on siderophore production. In addition, to confirm its QSI activity, we also examined its QS inhibition effect on *P. aeruginosa* PAO1 in the LB cultural medium. The results in **Figure 2B** show a white halo around HMMN.

### MICs and growth profiles

The MICs of hordenine and HMMN against *P. aeruginosa* PAO1 were 1,200 and 160 μg/ml, respectively. A concentration below the MIC was selected for the determination of the growth curve of *P. aeruginosa* PAO1 by hordenine (1,000, 800, 500, and 250 μg/ml) and HMMN (150, 130, 100, and 50 μg/ml) (**Figure S1**). The results showed that the selected concentration of hordenine had no effect on the growth of *P. aeruginosa* PAO1, and the selected concentration of HMMN of 150 μg/ml grew slowly in 5–20 h, but the density of cells after 20 h was very close to the density of DMSO in the blank group. It can be considered that 150 μg/ml HMMN does not affect the growth of *P. aeruginosa* PAO1.

### Quantitative analysis for *P. aeruginosa* PAO1 siderophore

**Figure 3:**
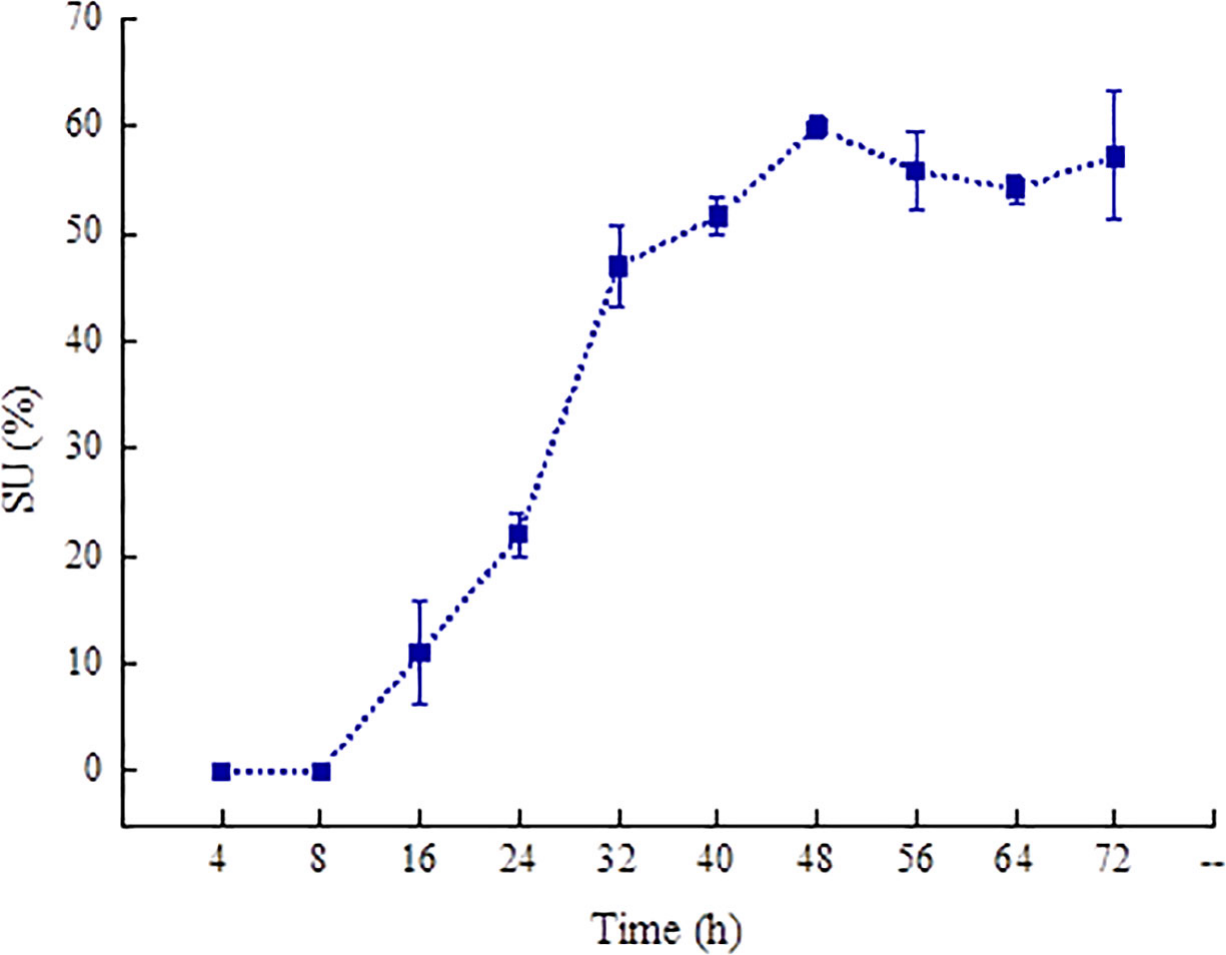
The content changes of siderophore produced in *P. aeruginosa* PAO1 with time.

Microorganisms can synthesize and secrete siderophores to fulfill the needs of their life activities. It can be seen from **Figure 1B** that *P. aeruginosa* PAO1 can produce siderophores. Therefore, we quantitated the siderophore content in *P. aeruginosa* PAO1 through the CAS detection solution. The siderophore content was denoted using the active unit for the siderophore *SU* value (**Figure 3**) (Xu et al., 2021). The content of the siderophore begins to accumulate from 8 h and reaches the maximum (59.84%) at 48 h in the *P. aeruginosa* PAO1 bacterial solution. After 48 h, the siderophore content gradually decreases.

### 2-Hydroxymethyl-1-methyl-5-nitroimidazole inhibits siderophore production of *P. aeruginosa* PAO1

**Figure 4:**
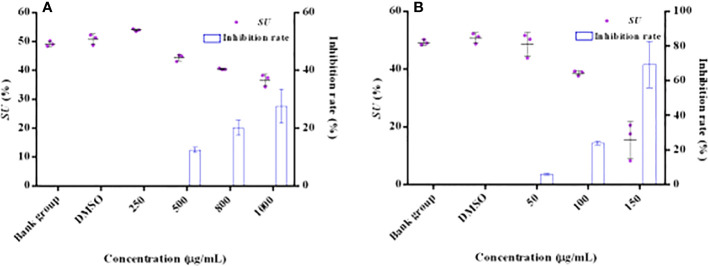
The effects of QSIs on the content of siderophore in PAO1. The influence and inhibition rates (%) of hordenine on the siderophore amount **(A)**; The influence and inhibition rates (%) of HMMN on the siderophore amount **(B)**.

The effect of HMMN and positive control hordenine on the siderophore content is shown in **Figure 4**. It showed that the siderophore contents decreased with hordenine concentrations increasing (250, 500, 800, and 1,000 μg/ml) in *P. aeruginosa* PAO1 (**Figure 4A**). It also revealed that the inhibition rate was 0.00%, 12.49%, 20.18%, and 27.65% for siderophores with hordenine treatment at 250, 500, 800, and 1,000 μg/ml, respectively. The maximum inhibition rate is 27.65% at 1,000 μg/ml of hordenine. In addition, **Figure 4B** presents the influence for different concentrations (50, 100, and 150 μg/ml) of HMMN on the siderophore production. The inhibition rates of HMMN at 150, 100, and 50 μg/ml are 69.37%, 23.95%, and 5.98%, respectively, for *P. aeruginosa* PAO1 siderophore content.

### 2-Hydroxymethyl-1-methyl-5-nitroimidazole occulted QS systems of *P. aeruginosa* PAO1

The QS is a system of cell–cell communication among bacteria. It can initiate the expression of related genes to regulate the bacterial population behavior, such as biofilm formation and the expression of virulence factors (Ilbert and Bonnefoy, 2013). The current study found that the siderophore inhibitor HMMN can occlude the QS system to inhibit biofilm formation and pyocyanin (a virulence factor) production.

#### The biofilm inhibition of 2-hydroxymethyl-1-methyl-5-nitroimidazole

**Figure 5:**
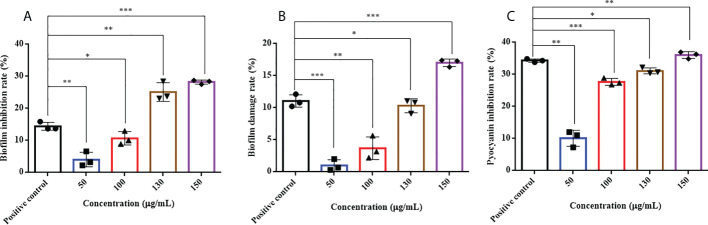
The effects of HMMN on biofilm and virulence factor pyocyanin in *P. aeruginosa* PAO1. the inhibition rates (%) of biofilm **(A)**; The damage rates (%) of biofilm **(B)**; The inhibition rates (%) of pyocyanin **(C)**. *p < 0.05, **p < 0.01, and ***p < 0.001.

HMMN and the positive-control hordenine have inhibitory effects on *P. aeruginosa* PAO1 biofilm formation (**Figure 5A**). It showed that different concentrations of HMMN can inhibit biofilm formation. The inhibition rates of biofilm formation are 4.03%, 10.67%, 25.11%, and 28.24% at concentrations of 50, 100, 130, and 150 μg/ml, respectively. The biofilm inhibition of *P. aeruginosa* PAO1 exhibits 14.42% at 500 μg/ml for the positive control.

#### The biofilm disruption of 2-hydroxymethyl-1-methyl-5-nitroimidazole

The erasure ability against the formed biofilm for *P. aeruginosa* PAO1 was determined at different concentrations of HMMN. As shown in **Figure 5B**, HMMN exhibited the ability to erase the formed biofilms at 50, 100, 130, and 150 μg/ml. The erasure rate for the formed biofilm is only 1.01% at 50 μg/ml, while it reaches 17.03% at 150 μg/ml. The erasure rate of the positive control hordenine is 11.05% at 500 μg/ml.

#### The influence of 2-hydroxymethyl-1-methyl-5-nitroimidazole on pyocyanin

Pyocyanin is a vital virulence factor for infection and biofilm formation in *P. aeruginosa* PAO1 (Retamal-Morales et al., 2021). It can be seen that HMMN can inhibit pyocyanin production at different concentrations (**Figure 5C**). The inhibition rates are 10.12% and 36.05% at 50 and 150 μg/ml, respectively. The positive control hordenine shows 34.35% inhibition at 500 μg/ml.

### 2-Hydroxymethyl-1-methyl-5-nitroimidazole downregulated *P. aeruginosa* PAO1 quorum sensing– and siderophore-related gene transcription

qRT-PCR analysis was carried out to evaluate the efficiency of HMMN on the expressions of QS-related genes (*las*I, *las*R, *rhl*I, *rhl*R, *pqs*H, and *pqs*R) and siderophore-related genes (*fur, sod*B, *mvf*R, *bfr*B, *ton*B1, *pch*G, and *fox*A) in *P. aeruginosa* PAO1.

**Figure 6:**
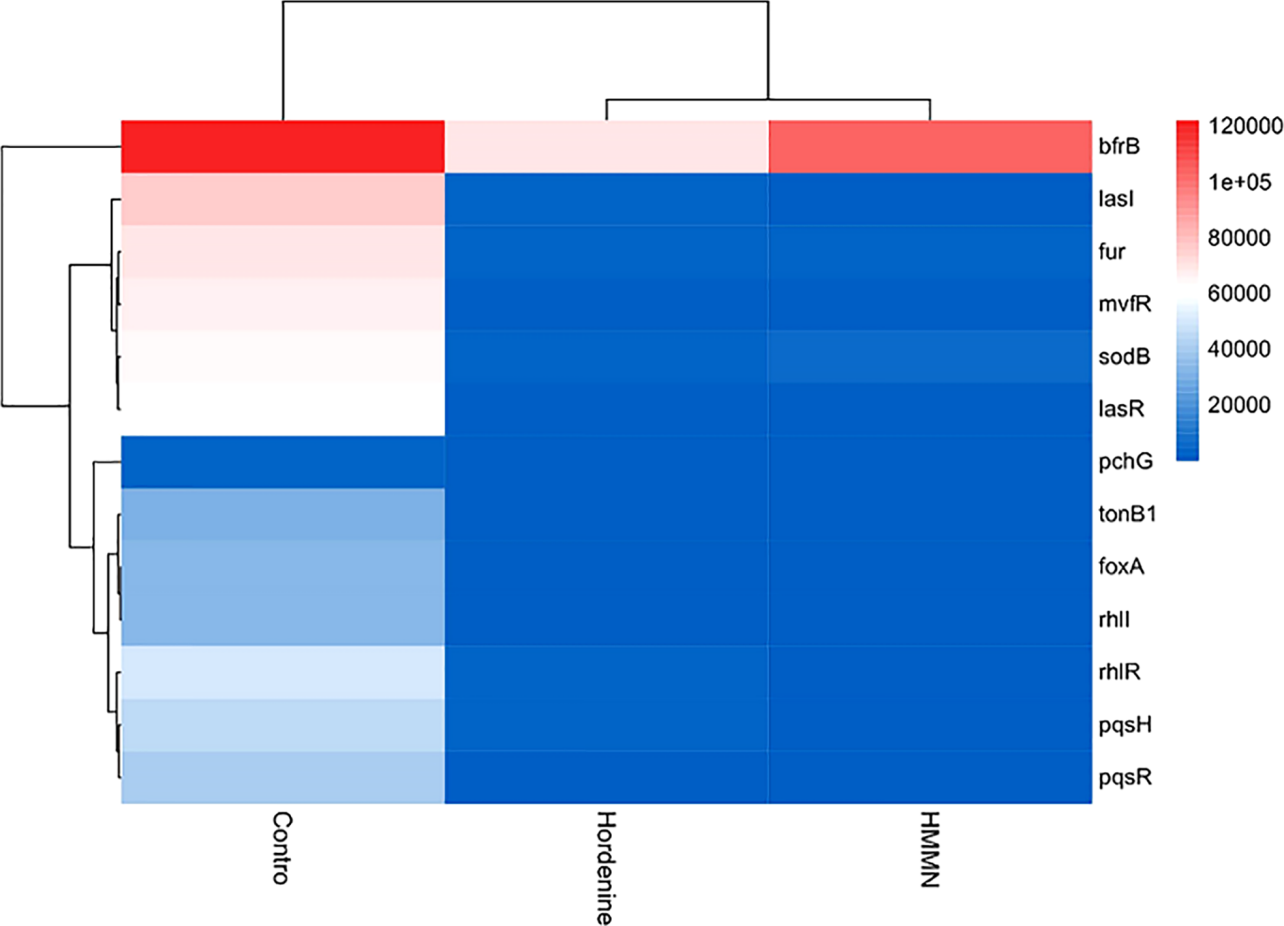
The hierarchical clustering maps. The hierarchical clustering maps in different treatments (hordenine and HMMN). The red side means upregulation, and the blue side means downregulation.

The heatmap in the current study showed QS- and siderophore-related gene expression (**Figure 6**). In addition, the gene expression was quantified after hordenine and HMMN treatment (**Figure S1**). As shown in **Figure 6**, the heatmap can be divided into three parts, with the leftmost part being the control. Hordenine and HMMN treatments were split into two parts. The color block in the heatmap is blue, indicating both drug treatment-downregulated expression for QS and siderophore-related genes, which is consistent with the results of the quantitative analysis in **Figure S1**. Hordenine can inhibit the expression of siderophore-related genes in *P. aeruginosa* PAO1 at 1,000 μg/ml, and HMMN can also inhibit the expression of QS-related genes at 130 μg/ml. Therefore, HMMN can be considered a QSI. Furthermore, the pink color was also interspersed in the hordenine treatment group. This may be due to the fact that gene expression differs in each of the samples treated differently. **Figures 6** and **S1** illustrate that hordenine and HMMN decreased the expression of QS- and siderophore-related gene expression. Hordenine can downregulate siderophore-related gene expressions (*fur, sod*B, *mvf*R, *bfr*B, *ton*B1, *pch*G, and *fox*A), and the inhibitor screened by the CAS method can also decrease the expressions of the QS-related genes (*las*I, *las*R, *rhl*I, *rhl*R, *pqs*H, and *pqs*R).

## Discussion

An increasing amount of evidence has shown that iron is an essential nutrient factor for microorganisms, but its low solubility under physiological conditions makes it challenging to utilize in bacteria (Balado et al., 2018). Therefore, pathogens have successfully evolved siderophores to obtain iron from the host, which can fit their growth and metabolism requirements (McRose et al., 2018). Previous studies showed that siderophore was essential for the pathogenicity in bacteria, and it was also one virulence factor (Zhou et al., 2018). It was found that QS and siderophores were mutually regulated (Persson et al., 2004). QS systems interact with iron and jointly regulate the expression of QS-related genes. McRose et al. reported that many bacteria could regulate the siderophore production by using the QS and found that *Vibrio harveyi* has an Fe- and QS-suppressed “two-for-one” siderophore gene cluster that releases both cell-associated and soluble products (Persson et al., 2004). In addition, iron regulation and QS systems in *P. aeruginosa* PAO1 work together to form a complex global network that regulates the expression of bacterial virulence factors and other physiological processes, affecting the process for bacterial infection.

QS regulates the siderophore production in many microorganisms. In addition, the siderophore content can be affected when the QS system is inhibited (Modarresi et al., 2015; Chowdappa et al., 2020). Therefore, this study speculated that siderophore inhibition can block microorganisms from absorbing iron; thus, they cannot provide the conditions for bacterial growth to trigger QS. The current study preliminarily proved that the CAS method was feasible to screen siderophore inhibitors according to the results of **Figure 1**. Thus, the siderophore inhibitor (HMMN) was obtained by the CAS method. Subsequently, this work studied the influence of hordenine and HMMN for the siderophore content in *P. aeruginosa* PAO1. Hordenine is one reported QSI for *P. aeruginosa* PAO1, and HMMN is one siderophore inhibitor of *P. aeruginosa* PAO1 obtained by the CAS method. The siderophore content gradually decreased with increasing HMMN doses, and the same was true for hordenine. These results indicate that the siderophore content of *P. aeruginosa* PAO1 appears in the early logarithmic phase, reaches the maximum accumulation in the stable phase, and begins to decrease in the late stable phase, which is consistent with the results from Payne’s research (Jiang et al., 2019). The above results further illustrate that QSIs can also interfere with the siderophore activity correspondingly. The effect of HMMN on QS systems for the *P. aeruginosa* PAO1 suggests that the HMMN screened by the CAS method can inhibit biofilm formation, erase formed biofilm, and inhibit pyocyanin production, which indicates that the siderophore inhibitor is feasible to be used as QSIs.

The *P. aeruginosa* PAO1 has three main QS systems (Waters and Bassler, 2005). The *las*I*/las*R genes regulate biofilm formation in the QS system, while the *rhl*I*/rhl*R genes significantly affect biofilm formation more than *las*I*/las*R (Abisado et al., 2018). In addition, *fur* is an iron uptake regulatory protein gene, which can control metabolism and virulence in response to iron availability (Sass et al., 2021). *P. aeruginosa* PAO1 possesses two cytoplasmic SODs, namely Fe-SOD (*sod*B) and Mn-SOD (*sod*M), and *sod*B has a significantly protective role against Ultraviolet-C (UV-C) radiations in *P. aeruginosa* PAO1 compared with *sod*M (Kim et al., 2015). *mvf*R is an iron-sensitive trigger that controls the production of siderophores and can also regulate biofilm formation (Zhou et al., 2017). *bfr*B can command the BfrB protein, which, as an essential iron storage protein with an internal cavity, can reserve up to 3,000 Fe^3+^, and severe iron dysregulation can be caused by blocking the expression of the *bfr*B gene (Santos et al., 2020). The *ton*B1 gene regulates the TonB system, which provides energy for the transport of siderophores (Ghorbal et al., 2016). The *pch*G gene is shown to regulate the production of the siderophore pyochelin in *P. aeruginosa* PAO1, and this gene knockout resulted in the inhibition of pyochelin synthesis (Maura and Rahme, 2017). Furthermore, the *fox*A gene is a ferrioxamine receptor. It is involved in the transcription and expression of siderophore synthesis when *P. aeruginosa* PAO1 is under iron-restricted conditions (Soldano et al., 2020). The expression of QS-related genes has the same trends as that of siderophore-related genes. The expression of QS-related genes is also suppressed when the expression of siderophore-related genes is suppressed. These results of the heatmap further demonstrate that QS and siderophores are mutually regulated.

The current study data suggest that the siderophore inhibitor seems to be one potential QSI. That is, the CAS method of screening siderophore inhibitors may be used to screen QSIs.

The present study found that hordenine, one reported QSI, can interfere with siderophore production. In addition, we found that one siderophore inhibitor (HMMN) can also act as one potential QSI to inhibit the biological effect of QS regulation, such as inhibiting biofilm formation, erasing the formed biofilm, and inhibiting the production of pyocyanin in *P. aeruginosa* PAO1.

## Data availability statement

The original contributions presented in the study are included in the article/**Supplementary Material**. Further inquiries can be directed to the corresponding author.

## Author contributions

All authors listed contributed immensely to this study. LY performed experiments and analyzed the data. LY, J-SL and WS finished the writing of paper. WS gave the constructive discussion in this study. A-QJ conceived and designed the study. A-QJ polished and revised the paper. All authors contributed to the article and approved the submitted version.

## Funding

This study was funded by the grants from the National Natural Science Foundation of China (82160664) and the Natural Science Foundation of Hainan Province (221CXTD434).

## Conflict of interest

The authors declare that the research was conducted in the absence of any commercial or financial relationships that could be construed as a potential conflict of interest.

## Publisher’s note

All claims expressed in this article are solely those of the authors and do not necessarily represent those of their affiliated organizations, or those of the publisher, the editors and the reviewers. Any product that may be evaluated in this article, or claim that may be made by its manufacturer, is not guaranteed or endorsed by the publisher.
