# Supplementary material for: 2-Hydroxymethyl-1-methyl-5-nitroimidazole, one siderophore inhibitor, occludes quorum sensing in Pseudomonas aeruginosa
Source: Front Cell Infect Microbiol. 2022 Sep 8;12:955952. doi: 10.3389/fcimb.2022.955952 (PMC9497652; doi:10.3389/fcimb.2022.955952)
Supplement: Supplementary file 1 [file DataSheet_1.pdf]

2-hydroxymethyl-1-methyl-5-nitroimidazole, one siderophore inhibitior, occludes quorum sensing in *Pseudomonas aeruginosa*

Lujun Yin[1], Jun-Sheng Liu[1], Wang Shen[1], Ai-Qun Jia[1, 2]*

[1] School of Pharmaceutical Sciences, Key Laboratory of Tropical Biological Resources of Ministry of Education, Hainan University, Haikou 570228, China

[2] One Health Institute, Hainan University, Haikou 570228, China

Corresponding author: Email: ajia@hainanu.edu.cn, Tel: 86-898-66254967. Fax: 86-898-66254967

Table S1 PCR primers for RT-qPCR

Genes	Primer direction	Sequence (5’-3’)
lasI	Forward	GTGTTCAAGGAGCGCAAAGG
	Reverse	AACGGCTGAGTTCCCAGATG
lasR	Forward	TCGAACATCCGGTCAGCAAA
	Reverse	GTTCACATTGGCTTCCGAGC
rhlI	Forward	ATTCTGGTCCAGCCTGCAAT
	Reverse	GTCTCGCCCTTGACCTTCTG
rhlR	Forward	CGATGCTGATGTCCAACCCG
	Reverse	GCGTCGAACTTCTTCTGGATGT
pasH	Forward	GACGAGGAGGGCTTCGTAAC
	Reverse	GTCACCGATCAGCACTACCC
pqsR	Forward	AACATGTTCCTCCAGGTCAT
	Reverse	GTTGAGATTGAAGGCGATGT
fur	Forward	CCACATGGTCTGCGTCGATA
	Reverse	CGCACGTAGAGCACCAGATT
sodB	Forward	GCCTTACGAAAAGAACGCCC
	Reverse	CTCTTGCCTTCGAACTCGGT
mvfR	Forward	CCTCCGGTTCGATTTCCTCC
	Reverse	GCATGTAAGGGATCAGGCGA
bfrB	Forward	AAGGACATCCTGGAGTCGGA
	Reverse	CAGTCGTCTTCGTGCATGTG

tonB1	Forward	ATGTCGCCACAGCCTTCAC
	Reverse	CAGGGACTCGCTTTCGTCTC
pchG	Forward	ACCTTCTATCCGCAGTTGCC
	Reverse	GAGCAGGCTCAATGGCACTT
foxA	Forward	TACGACTACGTGGTGATGCG
	Reverse	TCGTACAACGGCTTCTTGCT

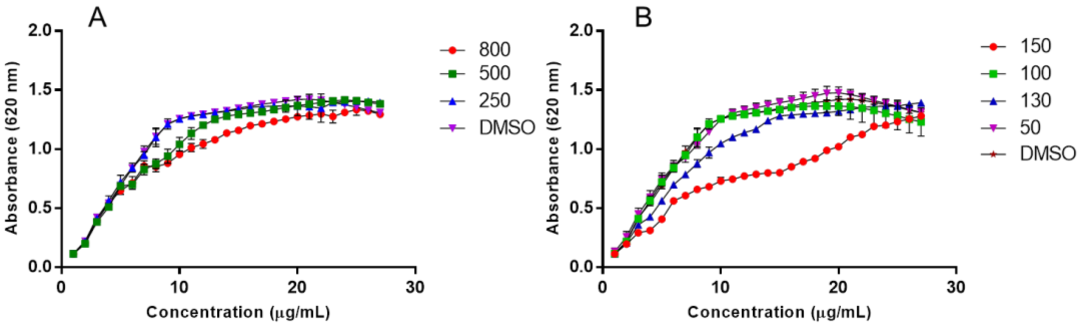

Fig S1 Growth curves of horderine (A) and HMMN (B) on PAO1

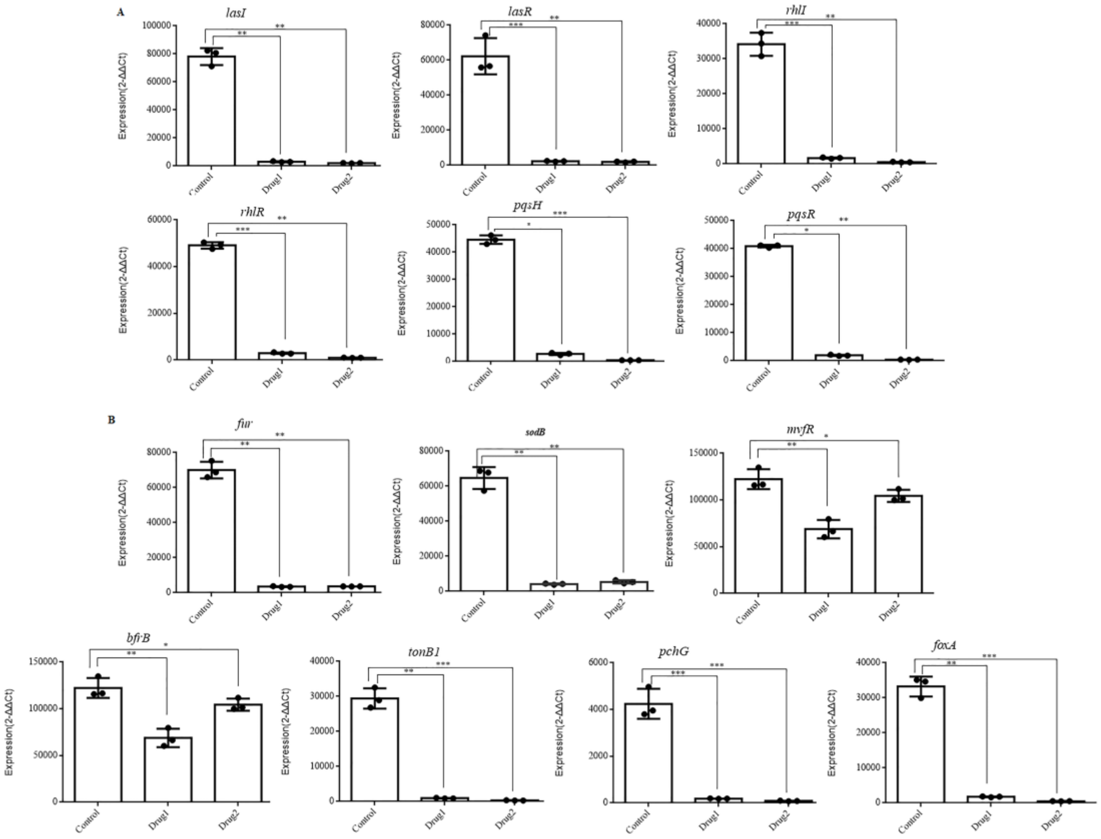

Figure S2 The influence of hordenine and HMMN on the expression of QS-related and siderophore-

related genes in PAO1. QS-related genes (A); Siderophore-related genes (B).
